# Quercetin and Related Chromenone Derivatives as Monoamine Oxidase Inhibitors: Targeting Neurological and Mental Disorders

**DOI:** 10.3390/molecules24030418

**Published:** 2019-01-24

**Authors:** Priyanka Dhiman, Neelam Malik, Eduardo Sobarzo-Sánchez, Eugenio Uriarte, Anurag Khatkar

**Affiliations:** 1 Faculty of Pharmaceutical Sciences, M. D. University, Rohtak 124001, India; priyankamdurtk@gmail.com (P.D.); neelammdurtk@gmail.com (N.M.); 2 Laboratory of Pharmaceutical Chemistry, Faculty of Pharmacy, University of Santiago de Compostela, Campus Vida, 15782 Santiago de Compostela, Spain; 3 Instituto de Investigación e Innovación en Salud, Facultad de Ciencias de la Salud, Universidad Central de Chile, 8370178 Santiago, Chile; 4 Departamento de Química Orgánica, Facultad de Farmacia, Universidad de Santiago de Compostela, 15782 Santiago de Compostela, Spain; eugenio.uriarte@usc.es; 5 Instituto de Ciencias Químicas Aplicadas, Universidad Autónoma de Chile, 7500912 Santiago, Chile

**Keywords:** monoamine oxidase, neurodegenerative disorder, mental disorders, quercetin, flavonoids, monoamine oxidase inhibitors, in-silico design

## Abstract

Monoamine oxidase inhibitions are considered as important targets for the treatment of depression, anxiety, and neurodegenerative disorders, including Alzheimer’s and Parkinson’s diseases. This has encouraged many medicinal chemistry research groups for the development of most promising selective monoamine oxidase (MAO) inhibitors. A large number of plant isolates also reported for significant MAO inhibition potential in recent years. Differently substituted flavonoids have been prepared and investigated as MAO-A and MAO-B inhibitors. Flavonoid scaffold showed notable antidepressant and neuroprotective properties as revealed by various and established preclinical trials. The current review made an attempt to summarizing and critically evaluating the new findings on the quercetin and related flavonoid derivatives functions as potent MAO isoform inhibitors.

## 1. Introduction

Depression and anxiety are estimated as incapacitating mental disorders which impose a huge health burden globally. According to the World Health Organization, major depression has now recognized as the fourth extensive cause of the worldwide in incapacity balanced life-years and could eventually turn into the second most critical cause by 2020 [1,2,3]. Treatment and therapies for mental disorder are also not economical. In the United States, the expenses of depression treatment and the costs experienced by less research work rate is estimated at more than $44 billion in 1990, which currently raised many fold [4,5]. Hence, the research for the discovery of potent and safe anti-depressant agents has attained importance due to a high mortality ratio of depressive disorders and their contribution for the destruction of other routine physiological processes.

Moreover, neurodegenerative disorders constitute the third most essential health issue in different developed countries. Alzheimer’s disease is the most widely recognized neurodegenerative disorder followed by Parkinson’s disease. Along with the aging problem of human society, Alzheimer’s disease (AD) has become one of the biggest threats to the modernize population. Alzheimer’s disease is indicated by nerve cells die in the cerebral cortex and accounts for 60 to 80 percent of dementia cases which affected more than 25 million people worldwide in 2000 and may eventually to increase to 114 million by 2050 [6,7,8,9]. General treatment of this disease is the utilization of dopaminergic agonists. Nonetheless, other medicinal options can be employed, like the utilization of specific monoamine oxidase B inhibitors, or the use of neuroprotective antioxidant agents to prevent the oxidative damage of neuronal cells. In the last few years, it has been proved that the overexpression of brain MAO-B also causes the neurodegeneration via generation hydroxyl radicals [10,11]. This certainty provokes an increase in free radical generation leads to oxidative stress, neuronal cell death and further the formation of the β-amyloid plaques [12].

The theory of MAO-B inhibitors for the prevention of neuronal damage is also accepted due to the reduction of hydrogen peroxide formation through inhibition of MAO. Clinical data suggests that patients with major depression have symptoms that are reflected changes in brain monoamine neurotransmitters, specifically serotonin (5-HT) and norepinephrine (NE) [13,14,15]. As per the most accepted hypothesis of depression, including the monoamine theory, dopamine (DA) is also implicated in the pathophysiology of various neurological disorders. Inhibitors of the enzyme monoamine oxidase were the first clinically used antidepressants; however, their utilization has reduced due to their documented serious adverse effects, their drug and food interactions, and the discovery of other target proteins [16,17,18,19]. Moreover, reports of hypertensive crises, liver toxicity, and hemorrhages and in some cases death resulted in the withdrawal of many MAO inhibitors from the market. Since then, medicinal chemists have been continuously involved in developing novel lead compounds that can selectively inhibit single isoform of MAO and can act as an effective therapeutic agent for various mental and neurological disorders [20,21,22]. 

Monoamine oxidase (MAO; EC 1.4.3.4) is a flavin adenine dinucleotide (FAD) dependent enzyme which is mainly localized on the outer mitochondrial membrane, responsible for the oxidative deamination of monoamines, including neurotransmitters, such as norepinephrine, dopamine, and serotonin (5-hydroxytryptamine [5-HT]) [23,24]. The two isoforms of MAO exists MAO-A and MAO-B, which differ in amino acid sequence, susceptibility to specific inhibitors, substrate specificity, and tissue distribution [25]. MAO-A preferentially deaminates noradrenaline and serotonin (5-hydroxytryptamine), whereas MAO-B preferentially deaminates β-phenyl-ethylamine and benzylamine. Inside the brain, MAO-B is mainly localized in the glial cells, while MAO-A found in the extraneuronal compartment and inside the dopaminergic, serotonergic and noradrenergic nerve terminals [26]. 

The oxidative deamination catalyzed through MAO leads to the formation of hydrogen peroxide (H_2_O_2_) and different reactive oxygen species has sufficient deleterious reactivity which accounts for associated health-related problems including neurological damage. The generation of H_2_O_2_ via MAOs is also reported to be a cytotoxic factor involved in oxidative stress, causes degeneration of nigral cells in Parkinson’s disease [27,28].

The modern search in the anti-MAO field is now directed toward the hybrid compounds, the latent risks in bioavailability and safety is a big concern in their further development. Despite notable progress in understanding their isoforms with respect to their 3D-structures, functionality, inhibitors, and substrates, no general rules have been formulated for the rational design of efficient, selective and reversible MAO inhibitors [29,30]. The current review made an attempt to identify the MAO inhibition property of quercetin and related derivatives and establish the rational design of new MAOIs from this investigation.

## 2. Chemistry and Therapeutic Journey of Quercetin and Related Derivatives

Quercetin (3,3′,4′,5,7-pentahydroxyflavone) is the significant illustrative of flavonols, a subclass of flavonoids. Quercetin, a type of flavonoids called flavonols, has received significant consideration in view of its overwhelming existence in herbs and food [31,32]. The major sources of quercetin are fruits such as citrus, apples, cherries and berries, vegetables such as broccoli, onions, and beverages such as red wine and tea. Moreover, it has been likewise found in several therapeutic plants, for example, *Aesculus hippocastanum*, *Ginkgo biloba*, and *Hypericum perforatum*. Research interest for this flavonol derivative is because of its diverse range of biological properties [33,34]. 

**Figure 1:**
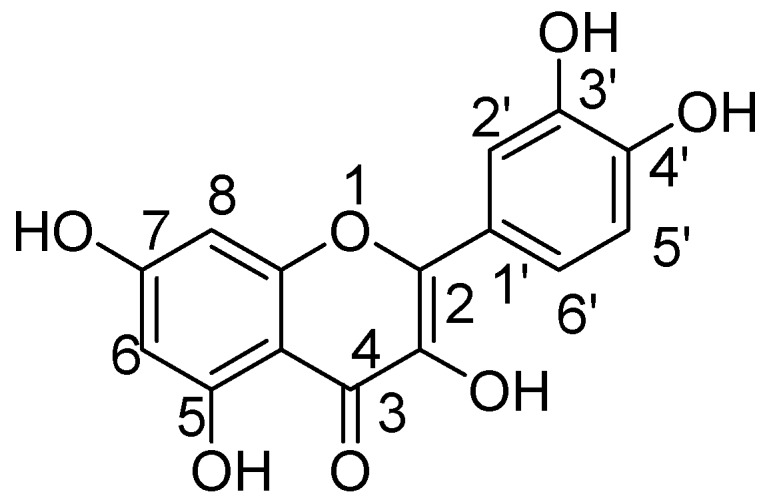
Quercetin.

Quercetin not only shows antioxidant activity like other natural flavonols but is also reported to have antiviral, anti-inflammatory, and antibacterial activities [35,36,37]. The exact mechanism by which quercetin shows these impacts are not completely clear, but rather it is conceivable that distinctive biochemical procedures are included. This natural flavonol is generally exists in a glycosylated form with its corresponding sugar part, generally glucose. The glycosylation may occur at any of the five OH groups of the flavonol ring, the most widely recognized quercetin glycoside exhibits the sugar moiety and structures speak to 60–75% of flavonoid intake [38]. Before oral ingestion, quercetin glycosides undergo deglycosylation either by cytosolic β-glucosidase or lactase phlorizin hydrolase. Further, the absorbed aglycone part is conjugated through sulphation, glucuronidation, or methylation. However, the aglycones and associated conjugates can cross the blood-brain barrier. Quercetin consists of a fused ring system with a benzopyran associated with an aromatic ring and phenyl substituents (Figure 1) [39].

**Figure 2:**
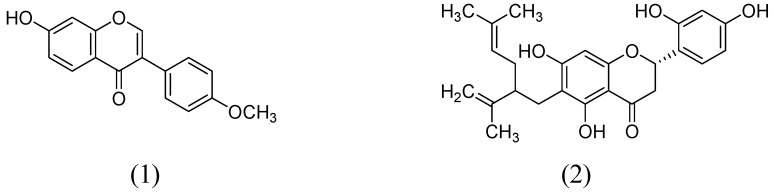
Structures of Formononetin (**1**) and Kushenol F (**2**).

In a study by Hwang and coworkers raveled the two natural flavonoids from the methanolic root extract of *Sophora flavecens*. The outcomes of the study indicated the dose-dependent MAO inhibition by kushenol F and formononetin with IC_50_ values of 69.9 and 13.2 µM, respectively (Figure 2). Interestingly, kushenol F mainly inhibited the MAO-B than MAO-A isoform shown the IC_50_ values of 63.1 and 103.7 µM, respectively. However, formononetin exhibited potential inhibitory effect towards MAO-B (IC_50_:11.0 µM) than MAO-A (IC_50_:21.2 µM) [40].

**Figure 3:**
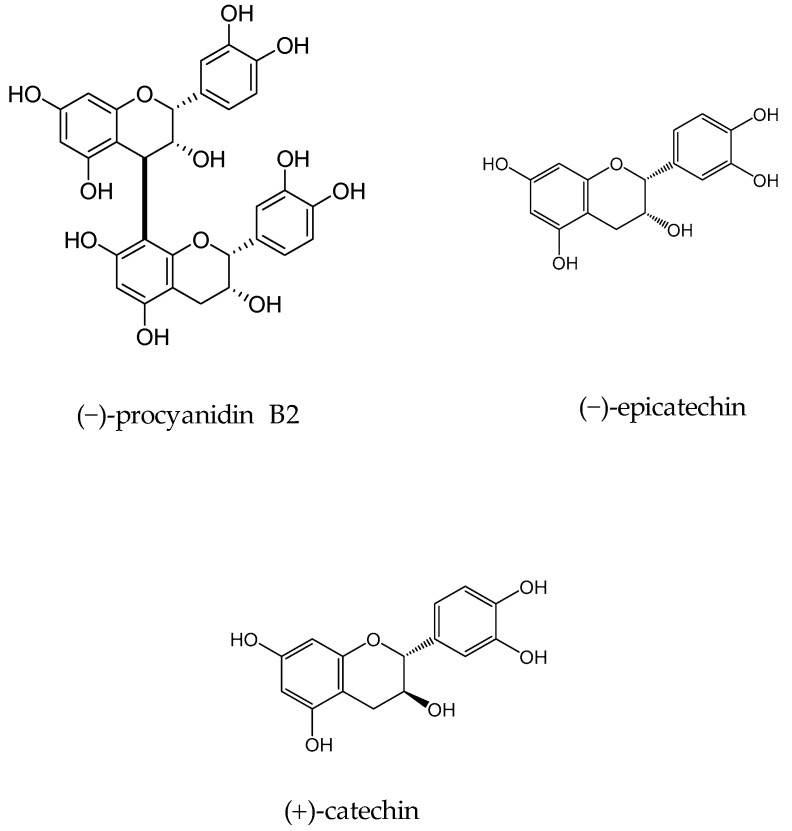
Structures of isolated constituents from *Banisteriopsis caapi*.

In 2010 Samoylenko and coworkers screened *Banisteriopsis caapi* (Malpighiaceae) constituents for the MAO inhibitory and antioxidative potential (found in South American liana of the family Malpighiaceae, *B caapi* is known to contain β-carboline alkaloids) [41]. Activity-guided fractionation of aqueous extract of *B. caapi* stems on led to the isolation of two popular proanthocyanidins (−)-procyanidin B2 and (−)-epicatechin (Figure 3). Epicatechin and (−)-procyanidin B2 showed considerable MAO-B inhibitory activity with IC_50_ 66 and 36 µM, respectively and very weak MAO-A inhibitory potential with IC_50_ 8.5 and 51.7 µM for procyanidin B2 and (−)-epicatechin, respectively. In addition, these components exhibited good antioxidant potential; both found to be more effective than standard antioxidants, vitamin C (IC_50_ < 0.14 and 0.58 µg/mL vs. 1.35 µg/mL), while (−)-epicatechin was found to be more active than Trolox (IC_50_ 0.14 µg/mL).

**Figure 4:**
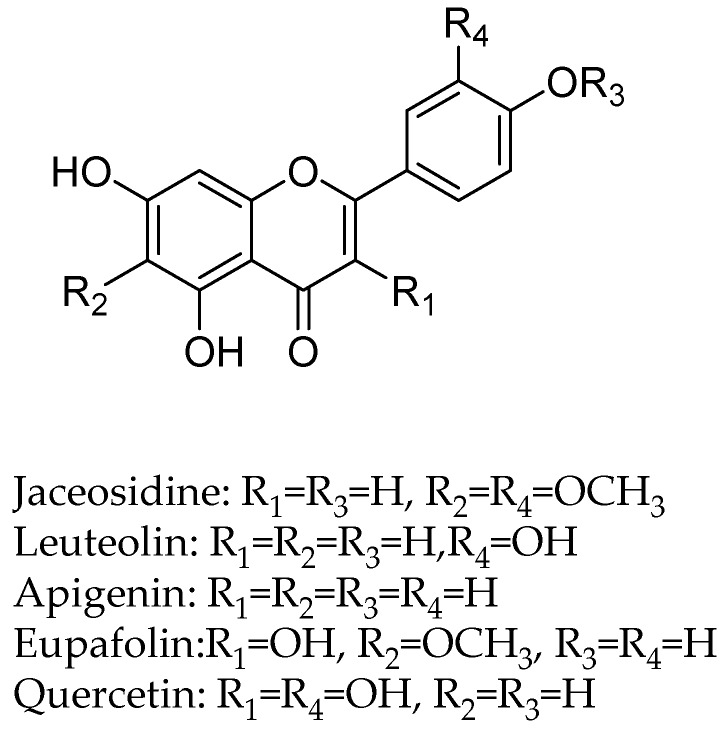
Flavanoids structures description.

In another study, the flavan-3-ols (−)-epicatechin and (+)-catechin were isolated from the hook extract of *Uncaria rhynchophylla* (Miq.) Jacks. using bioguided assay was found to inhibit MAO-B with the IC_50_ values of 57.9 and 88.9 µM, respectively, while the standard MAO-B inhibitor deprenyl showed an IC_50_ value of 0.3 µM [42]. (*U. rhynchophylla* (Rubiaceae), also known as cat’s claw herb, is a rhynchophylline plant species utilized in conventional Chinese medication). Lee et al., isolated flavonoids from 80% watery ethanol concentrate of entire plant of *Artemisia vulgaris* (Mugwort), and their structures were confirmed by utilizing different spectroscopic techniques. These compounds were recognized as jaceosidin, eupafolin, luteolin, quercetin, apigenin, aesculetin, esculetin-6-methylether, and scopoletin and were appeared to inhibit MAO with the IC_50_ estimations of 19.0, 25.0, 18.5, 72.9, 12.5, 1.0, 31.1, 32.2, and 45.0 µmol, respectively (Figure 4) [43].

**Figure 5:**
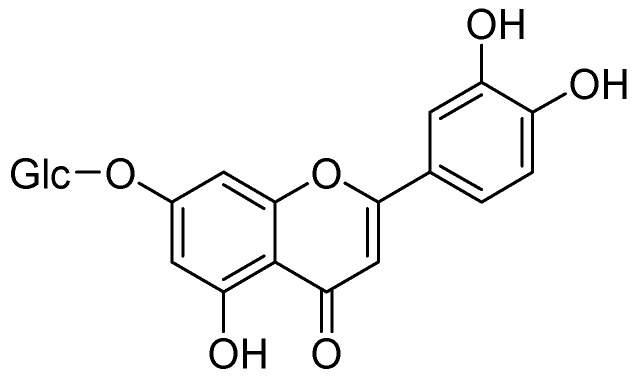
Cynaroside.

Conversely, Kim and coworkers isolated a flavonoid, cynaroside from *Angelica keiskei* Koidzumi (*A. keiskei* K.). Cynaroside showed notable MAO inhibition with IC_50_ values MAO-A400 µM and MAO-B 268 µM. Therefore, it is likely that that inhibition of MAO-B exerts antidepressant activity (Figure 5) [44]. 

**Figure 6:**
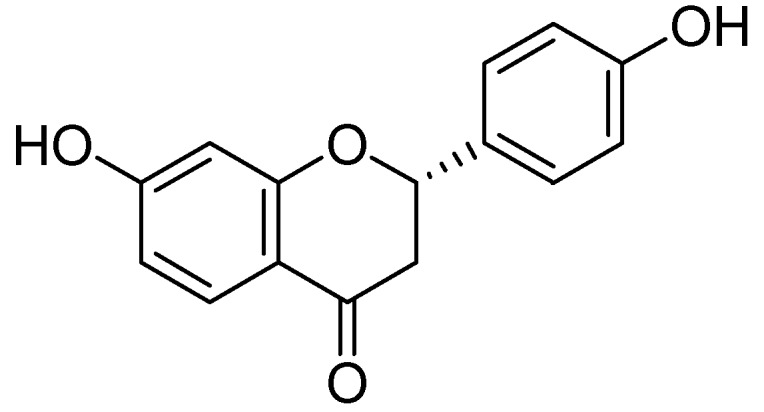
Liquiritigenin.

Another study by in 2000 by Pan and coworkers showed the MAO inhibition of isoliquiritigenin and liquiritigenin isolated from the methanolic extract of the flowering plant *Sinofranchetia chinensis* (Lardizabalaceae) was studied on rodent monoamine oxidase A and B [45]. MAO inhibitory activity was assessed radiochemically by using [14C] β-phenylethylamine (beta-PEA) and [14C]5-hydroxytryptamine (5-HT) as MAO-B or -A specific radio labeled substrates, respectively. Isoliquiritigenin and liquiritigenin acted as the potent MAO inhibitors against both MAO-B and -A in a dose-dependent manner (Figure 6). The MAO inhibitory IC_50_ values were calculated for isoliquiritigenin and liquiritigenin were 14 (12.8–15.6) and 32 (26–36) µmol/L for MAO-A isoform, 47.2 (39.5–54.5) and104.6 (89.0–118.9) µmol/L for MAO-B isoform, respectively. 

**Figure 7:**
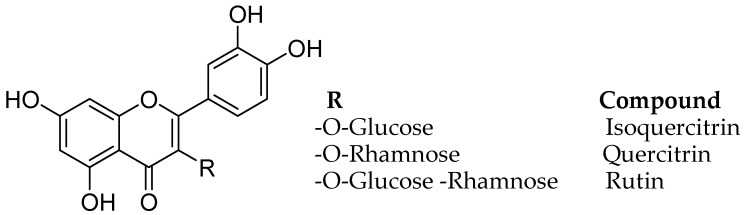
Structures of flavonoids.

Monoamine oxidase B inhibitory and free radical scavenging activities were evaluated for quercetin, rutin, isoquercitrin, and quercitrin, from the leave isolates of the *Melastoma candidum* (Melastomataceae) D. Don. using bioassay-guided fractionation (Figure 7) [46]. *Melastoma candidum* is a Chinese herb reported to clean heat and toxins, activating the blood and eliminating stasis, actuating the blood and wiping out stasis, for treating traumatic wounds, and for enacting fundamental vitality. The IC_50_ estimation of the four natural flavonoids, quercetin, rutin, isoquercitrin, and quercitrin on MAO-B was found ass 10.89, 3.89, 11.64, and 19.06 µM and analysis of enzyme kinetics calculated apparent inhibition constants (K_i_) of 7.95, 1.83, 2.72, and 21.01 µM, respectively. 

The in-vitro MAO inhibition by leaf extract of *Ginkgo Biloba* was carried out on mouse brain or liver monoamine oxidase (MAO)-A and -B activity [47]. The flavones apigenin and chrysin and the flavonols kaempferol and quercetin were extracted from a validated *Gingko biloba* preparation by reverse-phase HPLC system. All isolated flavonoid derivatives were observed as selective MAO-A inhibitors with the IC_50_ estimations of quercetin (4 µM), apigenin (2 µM), kaempferol (0.8 µM), and chrysin (1 µM). In the same assay phenelzine (irreversible and non-selective inhibitor of MAO) was taken as a reference compound (IC_50_ value 0.05 µM). 

**Figure 8:**
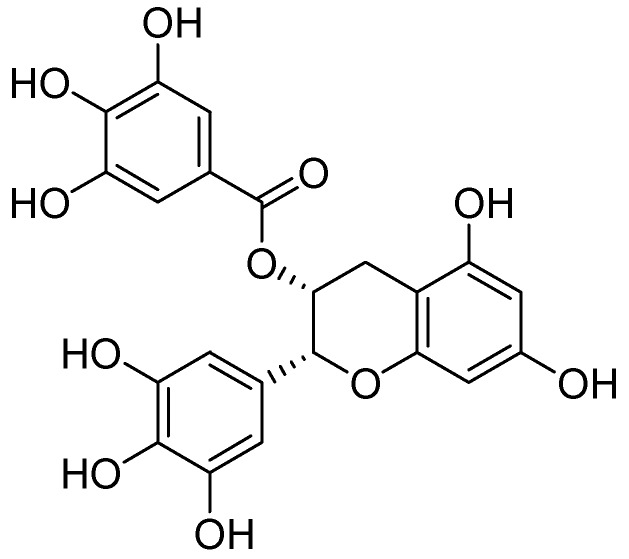
Epigallocatechin gallate.

Quercetin was isolated from the methanolic extract of heather (*Calluna vulgaris* (L.) Hull–Ericaceae) and was evaluated for MAO inhibition [48]. By exhibiting IC_50_ value of 18 µM quercetin was distinguished as a selective MAO-A inhibitor. However, clorgyline, an MAO-A selective inhibitor, showed an IC_50_ value of 0.2 µM in the same assay. Bio-guided fractionation of the *Rhodiola rosea* L. (Crassulaceae) prompted to the isolation of epigallocatechin gallate (EGCG) dimer (Figure 8) which was tested for MAO inhibition. It showed a sigmoidal dose-response curve for MAO-B with pIC_50_ of 4.74 µM, whereas l-deprenyl showed the pIC_50_ value of 7.24 for MAO-B inhibition [49].

**Figure 9:**
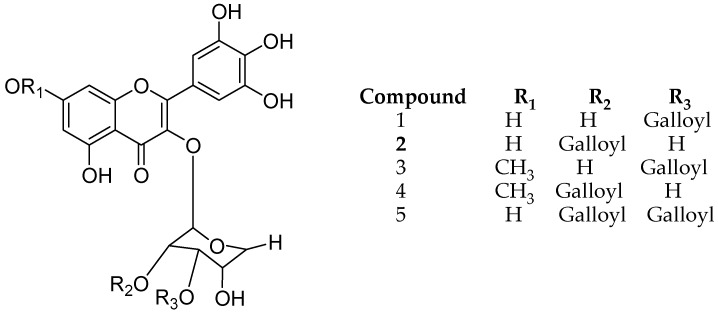
Structure description of myricetin galloylglycosides.

Lee and coworkers isolated different structure analogues of myricetin galloylglycoside from leaves of *Acacia confuse*, namely myricetin 3-*O*-(3″-*O*-galloyl)-d-rhamnopyranoside (**1**), myricetin 3-*O*-(2″-*O*-galloyl)-d-rhamnopyranoside (**2**), 3-*O*-(3″-*O*-galloyl)-Drhamnopyranoside 7-methyl ether1(**3**), myricetin 3-*O*-(2″-*O*-galloyl)-d-rhamnopyranoside 7-methyl ether (**4**), myricetin 3-*O*-(2″, 3″-di-*O*-galloyl)-d-rhamnopyranoside (**5**) (Figure 9). All five derivatives were evaluated for (semicarbazide-sensitive amine oxidase) SSAO inhibition and they all showed considerable amine oxidase inhibitory activity. Notably, the gallic acid at R_3_ position plays an important role for both biological activities [50].

**Figure 10:**
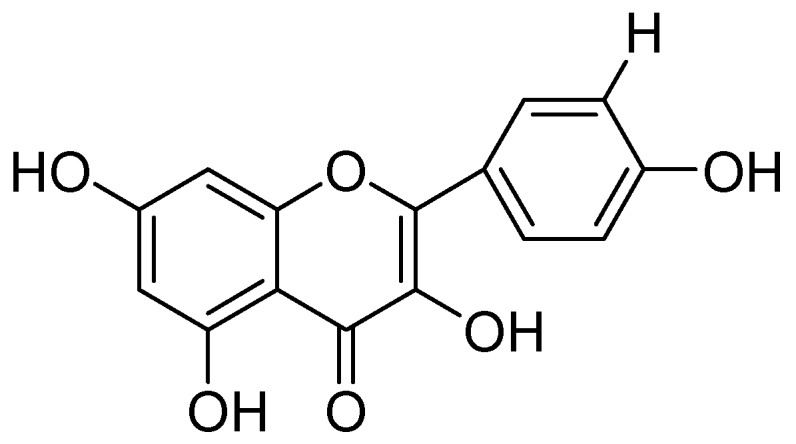
Kaempferol.

The neurological and neuroprotective properties of *Melissa officinalis* was also documented by Lopez and coworkers. They assessed MAO-A inhibitory potential of methanolic extract of *Melissa officinalis* the plant. The IC_50_ estimations for MAO-A by methanolic extract (19.3 ± 2.3) was found to be better than the aqueous extract (48.3 ± 5.7) [51]. The antidepressant action of *Morinda citrifolia fruit extracts* was evaluated by estimation of MAO inhibition studies [52]. The bioactivity-fractionation led two flavonoids, quercetin, and kaempferol. Bioassay of kaempferol (Figure 10) and quercetin, for MAO-A, calculated IC_50_ values of 3.15 M and 0.72 M, and 20.4 M and 31.7 M for MAO-B, respectively, selectivity indices for MAO-A shown as 28 and 10. 

**Figure 11:**
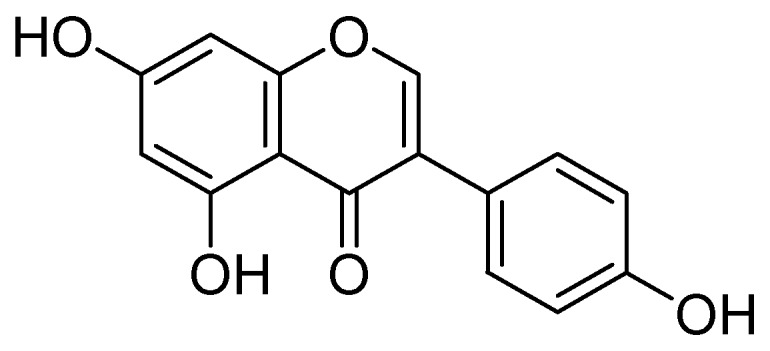
Genistein.

Isolation of kaempferol and apigenin flavonoids from *Sophorae flos* and demonstration of their strong MAO-A inhibitory effects over rat brain mitochondrial monoamine oxidase MAO-A with an IC_50_ estimation of 10, and 14 µM were carried out by Ryu and coworkers [53]. They concluded that both compounds do not preferentially inhibit MAO-B. Moreover, several other isoflavonoids were isolated from *Glycine max.* and screened. In which the genistein (Figure 11) selectively inhibited rat brain mitochondrial MAO-A with IC_50_ value of the 40 µM.

**Figure 12:**
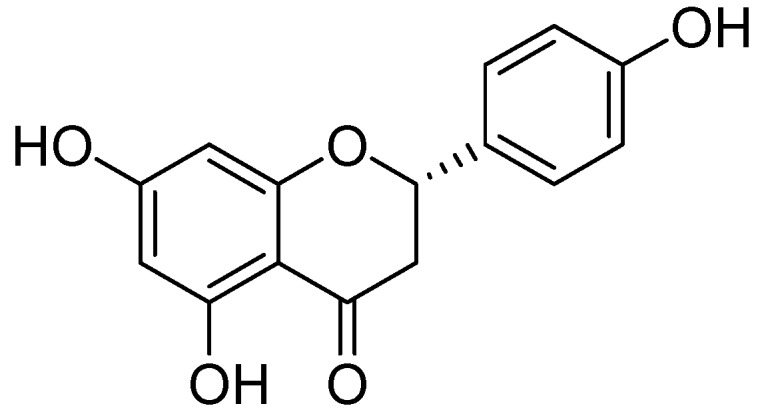
Naringenin.

Naringenin (Figure 12) was collected from the ethanolic extract of *Mentha aquatica L*. via by bioactivity-guided fractionation on preparative TLC [54]. The MAO inhibitory IC_50_ values by naringenin were calculated as 340 ± 30 M for the homogenate of rat liver mitochondrial fraction, 288 ± 18 M was calculated for MAO-B and for MAO-A 955 ± 129 M. However the MAO inhibitory potential of was not more than quercetin.

**Figure 13:**
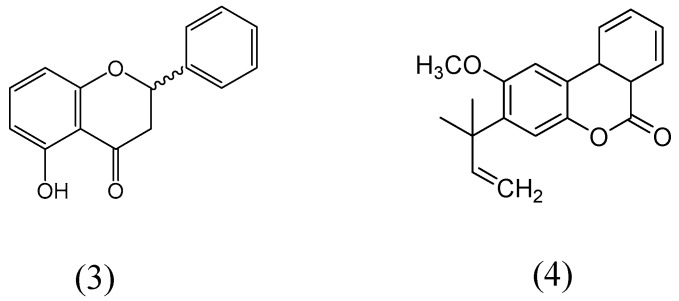
Structures of 5-hydroxyflavanone (**3**) and 2-methoxy-3-(1-dimethylallyl)-6a,10a-dihydrobenzo(1,2-c)chroman-6-one (**4**).

5-Hydroxyflavanone and 2-methoxy-3-(1-dimethylallyl)-6a,10a-dihydrobenzo(1,2-c)chroman-6-one (Figure 13) were extracted from the dried bark methanolic concentrate of *Gentiana lutea* [55]. Monoamine oxidase activity was evaluated on rat brain mitochondria fraction. Compound 2-methoxy-3-(1-dimethylallyl)-6a,10a-dihydrobenzo(1,2-c)chroman-6-one specifically inhibited MAO-B isoform, whereas entire inhibition was observed at 9 µM. 5-hydroxyflavanone exhibited more affinity for MAO for MAO-B than MAO-A isoform. Enzyme kinetics for the MAO inhibition was carried out by Lineweaver-Burk plots and both compounds showed the reciprocal plot curves for MAO inhibition activities, where the concentration of substrate also found intersected to the ordinate. The apparent Ki values of compounds of 5-hydroxyflavanone and 2-methoxy-3-(1-dimethylallyl)-6a,10a-dihydrobenzo(1,2-c)chroman-6-one for MAO-B were calculated as 1.1 µM and 1.4 µM, respectively.

In another study MAO inhibition studies were performed on pure anthocyanidins and the MAO-A and MAO-B inhibitory IC_50_ values were calculated as for pelargonidin (28 µM and 45 µM), peonidin (41 µM and 25 µM), malvidin (32 µM and 20 µM), delphinidin (36 µM and 38 µM), cyanidin (31 µM and 33 µM), and petunidin (35 µM and 45 µM).

Furthermore, the various diglycosides and glycosides of the above- revealed anthocyanidins were also investigated for MAO inhibition with IC_50_ estimation of 30–120 µM against MAO-A and 32–247 µM against MAO-B [56].

Bioactivity-guided isolation of seven flavonoids from the methanolic extract of *Cayratia japonica* was carried out to evaluate MAO inhibitory potential [57]. The structures of the components were identified as apigenin, apigenin-7-*O*-β-d-glucuronopyranoside, quercetin, luteolin, (+)-dihydro-kaempferol (aromadendrin), (+)-dihydroquercetin (taxifolin), and luteolin-7-*O*-β-d-glucopyranoside. Among the all titled compounds, flavonol, quercetin as well as the flavones such as luteolin and apigenin showed potential MAO inhibitory effects with estimated IC_50_ values of 33.7 μM, 23.7 and 6.7 respectively. Furthermore, quercetin was found as most active MAO-A inhibitor (IC_50_ value: 1 μM) than MAO-B (IC_50_ value: 90 μM), whereas luteolin and apigenin also mainly inhibited MAO-A isoform (IC_50_ values: 5.0 and 1.0 μM, respectively) as compared with MAO-B (IC_50_ values: 60.0 and 13.0 μM, respectively). Moreover, the flavanonol derivatives, aromadendrin, and taxifolin exhibited poor inhibition (IC50 values: 152.9 μM and 155.1, respectively). The flavone glycosides, luteolin-7-*O*-β-d-glucopyranoside and apigenin-7-*O*-β-d-glucuronopyranoside exhibited less MAO inhibitory activity (IC_50_ values: 118.6 and 81.7 μM, respectively).

Fourteen types of herbal plants were evaluated for MAO-B inhibitory potential. The extracts of *Chrysanthemum indicum*, *Sophora japonica*, *Artemisia Messer-Schmidtiana*, *Ericibe obtusifolis* significantly inhibited the MAO-B enzyme. Among them, *Chrysanthemi indicum* was selected for fractionation and identification of its active components, which led some flavonoids as diosmetin, acacetin, apigenin, 5,7-dihydroxy chromone, luteolin, and eriodictyol. The MAO inhibitory IC_50_ values for 5,7-dihydroxy chromone and diosmetin were calculated as following: 2.50, 0.20, 2.10 µM respectively, while the other principles showed weak inhibition [58].

**Figure 14:**
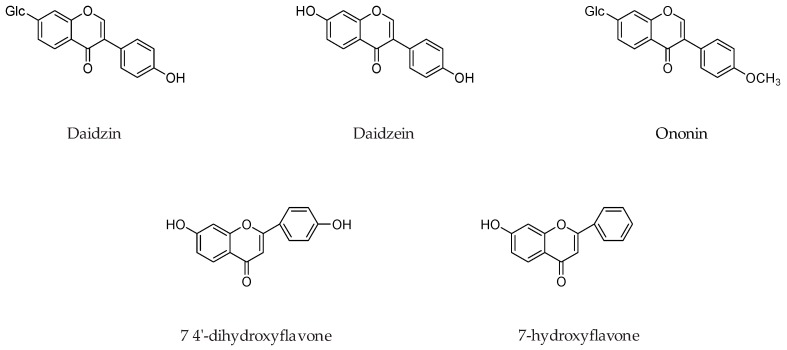
Chemical structures of isoflavone daidzein and its various analogs.

Isoflavone daidzein and its various analogs such as daidzin, ononin, 7-*o*-ω-carboxypentylflavone, 7-*O*-*r*-carboxyheptyldaidzein, 7-*o*-isopropyldaidzein, 7-*o*-dodecyldaidzein, 7-*o*-ω-carboxyheptyldaidzein, 7-*o*-ω-carboxyundecyldaidzein, 7-*o*-ω-hydroxyethyl-2-(2-oxyethyl) oxyethyldaidzein (Figure 14) were evaluated for MAO inhibition. It was concluded that presence of a free 4′-OH function on isoflavone ring and a straight 7-*O*-alkyl chain substitution, that has a terminal polar function such as -COOH, -OH, and -NH_2_ is crucial for MAO inhibition. Most preferable chain lengths for the MAO inhibition were 7-*O*-ω-hydroxy, 7-*O*-ω-carboxy, and 7-*O*-ω-amino substituent, were respectively [59]. 

## 3. Molecular Docking Studies of Quercetin and Related Flavonoid Derivatives

In 2006, Zhang et al. determined MAO inhibition of natural flavonoids by docking experiments. Docking methodologies revealed that the quercetin can thoroughly bind within the active site of the hMAO-B (with drug score of −61.5). Close inspection of the docking poses shown that the binding not only depends on the hydroxyl groups at 7th or 5th positions (that is equivalent to the positions 1st and 3rd in xanthone ring) but also on the OHs present at other positions (Table 1). Furthermore, the ring B in catechol seems very important which notably contributes for the binding, and could increase the MAO inhibiting capacity of quercetin. This special character of OH and ring B found unique as compared to xanthones, which do not require catechol for the better restricting association with MAO protein and can be justified to some degree by the more adaptability of flavonoids than xanthones. This may because of the ring B in flavonoids that is rotatable through C2-C10 bond, through so the flavonoids modify their conformational changes to tie inside the dynamic locales of MAO proteins, pictured by the superimposed quercetin adaptations in the coupling pocket of MAO-B [60].

**Figure 15:**
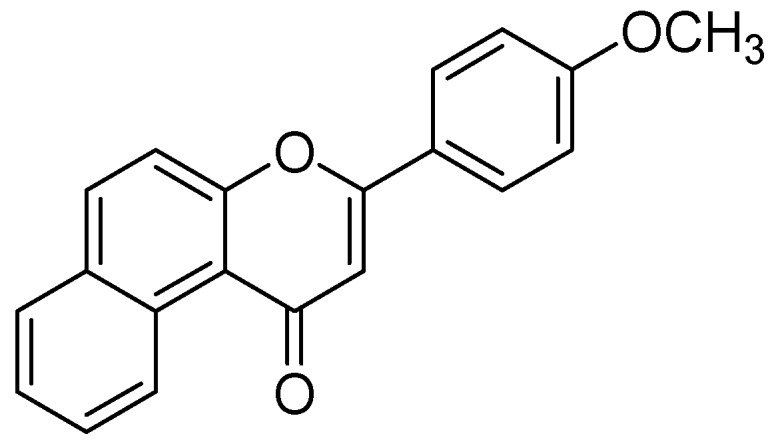
3-(4-methoxyphenyl)-1*H*-benzo[f]chromen-1-one

The methanolic extract from leaves of *Hypericum hircinum* showed monoamine oxidases (MAO) inhibition. The bioactivity guided isolation prompted to the isolation of quercetin and five different components, recognized for the first time from *H. hircinum* [67]. Quercetin was the main compound with a specific inhibitory action against MAO-A, with an IC_50_ estimation of 0.010 µM. To illustrate the behavioral impacts of quercetin the in-vivo animal study on mice was performed using the forced swimming test. The mechanism of inhibition was further confirmed by molecular docking studies by applying the graphical user interface by MacroModel (Maestro GUI), Schrodinger [61]. The interaction energy has shown a good correlation between the exploratory inhibition information and affirmed the particular MAO-A recognition in both configurationally ensembles calculated along with molecular docking and full energy minimization. The authors observed that quercetin associated well within the hMAO-A binding site than in the hMAO-B binding site due to the formation of maximum *π*-*π* interaction and intermolecular hydrogen bonds. Moreover Greeson and coauthors also discussed the pharmacological, toxicological, and clinical aspects on MAO inhibitory action of St. John’s wort (*Hypericum perforatum*) [62]. Design of the 3-(4-methoxyphenyl)-1H-benzo[f]chromen-1-one (Figure 15) was carried out MAO-A inhibitor PDB code (2Z5X) by in-silico techniques [68]. The strategy for the design was to modify the flavanone (C6-C3-C6 three ring skeleton) to benzoflavanone composes naphthalene in spite of the first C6 ring. The binding of this compound with MAO-A was investigated by molecular docking by FlexX program [69]. Visual inspection of the docking poses indicated four more residues, Tyr69, Gly67, Gly443, and Met350 in the complex of 3-(4-methoxyphenyl)-1*H*-benzo[f]chromen-1-one with MAOA. Moreover, 31 hydrophobic inactions were established with the MAO-A binding cavity. 

**Figure 16:**
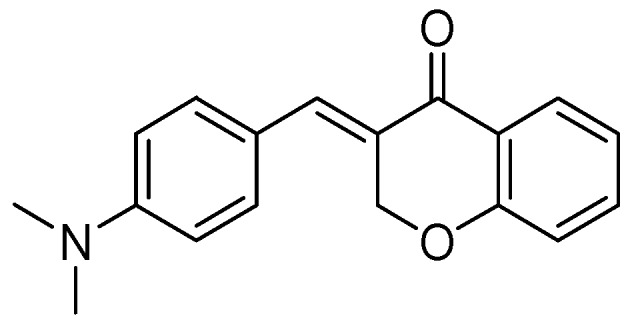
(*E*)-3-(4-(Dimethylamino)benzylidene)chroman-4-one.

**Figure 17:**
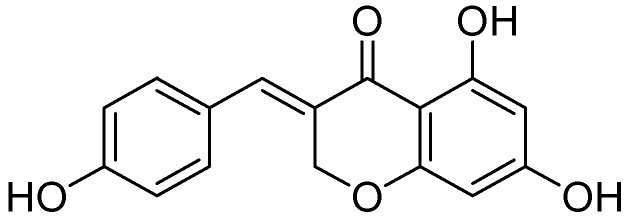
(*E*)-5,7-dihydroxy-3-(4-hydroxybenzylidene)chroman-4-one.

A series of homoisoflavonoids 3-benzyl-4*H*-chromen-4-ones, 3-benzylchroman-4-ones, and have been synthesized and benzylidenechroman-4-ones was investigated for in vitro as inhibition of h-MAOA and h-MAOB by Desideri et al. [70]. Rationalized docking studies provided the inhibitory affinity of homoisoflavonoids with respect to isoforms of hMAO-A PDB (2XFN) and hMAO-B PDB (2Z5Y). Visual inspection of the docking poses of the (*E*)-3-(4-(dimethylamino)benzylidene)chroman-4-one chromone (Figure 16) and (*E*)-5,7-dihydroxy-3-(4-hydroxybenzylidene)chroman-4-one (Figure 17), chromanone rings were positioned near to the flavin ring of hMAO-A. Interestingly, most of the docked ligands exhibited the same kind of binding interactions. The only difference in MAO-A PDB (2Z5Y) binding interaction was due to the presence of a hydroxyl OH in the former analog that established a single hydrogen bond with N5 atom of FAD. Moreover, (*E*)-5,7-dihydroxy-3-(4-hydroxybenzylidene)chroman-4-one, nearby the FAD cofactor, formed an exclusive bond with Tyr69. The (*E*)-3-(4-(dimethylamino)benzylidene)chroman-4-one was found to be involved in hydrophobic interactions with Tyr444 and Asn181. 

Furthermore, Chimenti et al. [71] reported another series of synthetic flavanones, thioflavones, and flavones, analogs, active against both monoamine oxidase isoforms (MAO-A and -B). To visualize the binding mechanism of both isomers of (*R*)-2j and (*S*)-2j enantiomers, docking studies were carried out by the Glide with respect to both isoforms of hMAO [61]. The molecular modeling studies showed good correlations to the experimental results, and hence proved the conformational flexibility of both 3 dihydrochromen-4-one, 2-(4-fluorophenyl)-7-methyl-2 enantiomers to fit within the active site of both hMAO isoforms with characteristic affinity. The most active compound 2-(4-fluorophenyl)-7-methyl-2,3-dihydrochromen-4-one exhibited nanomolar the inhibitory potential as the racemate and was the most potent inhibitor in the two enantiomeric forms. 

More recently Turkmenoglu et al. [72] evaluated the hMAO inhibitory activity of four flavonoids, isoscutellarein 7-*O*-[6′′′-*O*-acetyl-β-d-allopyranosyl-(1→2)]-6″-*O*-acetyl-β-d-gluco-pyranoside, xanthomicrol, isoscutellarein 7-*O*-[6′′′-*O*-acetyl-β-d-allopyranosyl-(1→2)]-β-d-gluco-pyranoside and salvigenin from *Sideritis* using recombinant hMAO isoenzymes. Docking experiments showed salvigenin as the most potent hMAO-A inhibitor by forming several van der Waals and electrostatic interactions within the active site of the hMAO-A where aromatic coumarin ring of the salvigenin established two π-π staking with TYR444 and TYR407 residues situated in the cavity. Moreover, the xanthomicrol has shown selective inhibitory interactions towards hMAO-A by forming five hydrogen bonds with the amino acids residues of the side chains of active site hMAO-A isoform (between the hydroxyl and GLY66, hydroxyl and ASN181, hydroxyl and LYS305 and methoxy and TYR444). The aromatic coumarine ring of xanthomicrol was observed sandwiched between the TYR407and TYR444 amino acid residues, which established two π-π interactions with TYR407 and formed a hydrophobic cage within the binding pocket. More relevant molecular binding interactions among the natural leads and hMAO the docked complexes were analyzed by 2-dimensional methods. The docking profile of the selected compounds is given in the (Table 2).

In a subsequent paper, Gao et al. [63] reported a magnificent *in silico* target fishing protocol based on mining of diverse database, molecular modeling, ligand similarity searching, structure-based pharmacophore searching and docking protocols together for searching new potential therapeutic anti-Parkinson agents. They concluded that the establishment of productive enzyme-inhibitor interaction behavior of top two ranked targets monoamine oxidase B (MAO-B) and catechol-O-methyltransferase (COMT) from the seven selected protein targets as important targets for baicalein function by literature. For the study flavonoid, baicalein was isolated from the root extract of *Scutellaria baicalensis Georgi*. Docking calculations were carried out using Glide software for the comparison of binding energy of baicalein with the standard [61]. Two catecholic OH groups of baicalein showed hydrogen bonding with Leu167and Leu164, respectively. Moreover, a network of productive hydrophobic interactions also appeared between MAO-B and baicalein, which appreciably contributed to the binding interactions. Baicalein notably reduced the formation of intracellular NO (nitric oxide), reactive oxygen species, and extracellular NO, due to reduced cell death, exposure of NMDA (*N*-methyl-d-aspartic acid). It was noticed that NMDA receptor with generally low agreement score cannot be a valuable target for baicalein, having no inhibitory impact on [3H]MK-801 binding. The authors validated and developed a consensus scoring formula for ranking of the targets of a titled compound.

Sivaraman and coworkers performed docking calculations to rationalize the MAO inhibitory potency of luteolin, quercetin, kaempferol, and apigenin by using Auto Dock tools [64]. The binding free energy (ΔG) and inhibition constants (Ki) of the natural ligands were computed via the Lamarckian Genetic Algorithm (LGA) of AutoDock application. Perfect to good correlations were established between the experimental and calculated Ki values [73] (Table 3).

**Figure 18:**
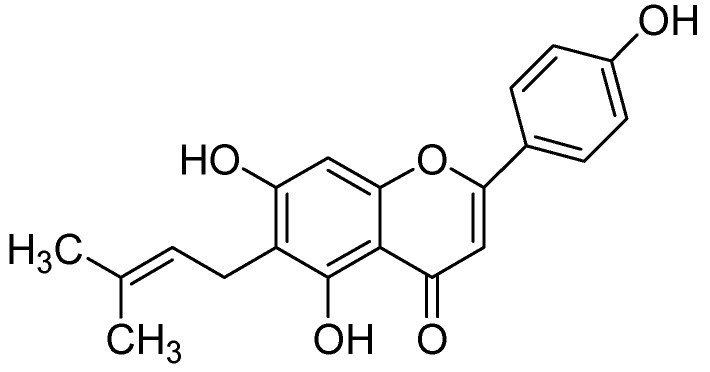
6-Prenyl apigenin.

In a later work, Beula and coworkers [65] isolated 6-prenyl apigenin (Figure 18) from a methanolic extract of *Achyranthes aspera* seeds and computed molecular docking to get insight into the binding modes of 6-prenyl apigenin within the monoamine oxidase-A enzyme pocket. Molecular docking studies were carried out by using AutoDock [64], revealed 6-prenyl apigenin as a promising candidate for hMAO-A inhibition by exhibiting calculated inhibition constant of about 1.23 µM and docking score of −8.06. To understand the structural role of the isolated 6-prenyl apigenin the 3D structural was divided into three fragments so-called flavones skeleton, the phenolic group at the 2nd position of the nucleus and a distal side chain located at the 6th position. It is worthy to note that the π electrons of the hydroxyl groups were sandwiched between phenolic side chains of TYR407 and TYR 444 composed the ‘aromatic cage’ of the hydrophobic pocket of the enzyme. Furthermore, another π-π stacking interaction has appeared between flavone moiety and TRP 441 residue within the hMAO-A binding site. 

More recently Zarmouh et al. [74] reported the MAO inhibitory activity of the natural prenylflavanones, genistein (GST) and bavachinin (BNN) from the ethanolic extract of *Psoralea corylifolia* seeds. *Psoralea corylifolia* is a medicinal plant widely documented for its antiaging properties. These two unique prenylflavanones selectively inhibited MAO-B enzyme with the highest potential. Docking methodologies predicted the binding affinity for both flavonoids, genistein (GST) and bavachinin (BNN). Zarmouh and coworkers further explored their earlier studies in 2015 [75], the flavanone bavachinin (BNN) and its other structural analog bavachin (BVN) from the seeds of *Psoralea corylifolia* L. for their human MAO inhibition. Docking studies were performed to validate the correct binding and mechanistic insight into docking poses depicted in (Table 4). The docking poses were analyzed with reference of the bound ligands of the crystal structures of human MAOB-2-(2-benzofuranyl)-2-imidazoline complex and human MAO-A-harmine complex. 

The same group further studied the isoflavone genistein (GST) and its structural analog daidzein (DZ) as promising MAO-A and MAO-B inhibitors Zarmouh et al. [76]. Molecular docking studies of GST and DZ was performed within the binding pocket of MAO isoforms. In the case of the hMAO-B, both analogs chromone ring were docked entirely within the hydrophobic part of the binding site (substrate-binding domain). Due to their phenolic OH moiety near to the entrance cavity, both derivatives were positioned far from FAD and its surrounding tyrosine amino acid residues. The GST C_4_′-OH group moiety formed maximum hydrogen bonds far from the hydrophobic sites than DZ. This molecular network increased the reversibility due to not affecting the flavin structure and possessing reversible H-bond interactions and hydrophobic. In case of MAO-A, the chromone ring of two isoflavone ligands were positioned in the compact entrance cavity near the to the flavin cofactor (FAD), whereas their hydroxy-phenyl group was located to the hydrophobic active site entrance surfaces. Both isoflavones possessed crossed and similar orientation as compared with the standard. A best-matched docking pose of the standard was contributed by a slight pull of GST toward a hydrophilic zone at its C_5_-OH group. The docking studied observations are given in (Table 5).

Recently Gidaro and coworkers reported a computational method to generate the binding modes of quercetin and kaempferol to the active site of both hMAO isoforms [66]. All the lowest energy conformations were generated through the application of the OPLS-2005 force field, before docking simulations methods. Computation of free binding energy (ΔG Bind) for each docked complex was determined through Prime/MM-GBSA approach along with OPLS-2005 force field and the default parameters settings. Subsequently, quantum mechanics/molecular mechanics (QM/MM) docking calculations were carried out by the Schrödinger QM-Polarized Ligand Docking Protocol (QPLD) application [61]. Finally, results of molecular dynamic simulations established the specificity of the reversible inhibitors was mainly because of the structural shape and size of the substrate/inhibitor cavity, restricted by PHE208 and ILE335 amino acid residues within hMAOA, which correspond to ILE199 and TYR326 in hMAO-B. Binding mode of the kaempferol in the catalytic site of hMAO-A showed hydrophobic interactions with key residues of hMAO-A for a longer time than in the hMAO-B pocket. Kaempferol retained 90% of the simulation time with PHE208 and 80% of the total simulation time hydrophobic interactions with ILE335 of hMAO-A. Conversely, in the binding pocket of hMAO-B, kaempferol retained 80% simulation time with TYR326 and 30% of the simulation time through hydrophobic contacts with ILE199. The detailed description of docking analysis is given in (Table 6).

## 4. Conclusions

**Figure 19:**
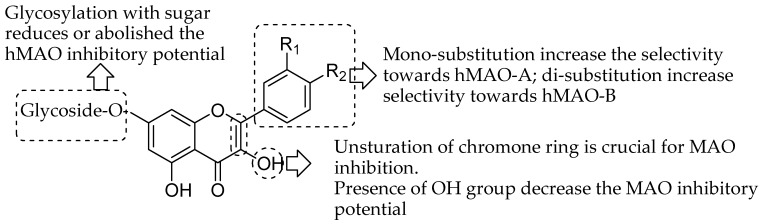
Structure-activity relationships (SAR) trends inferred from the data of enzymatic and docking experiments reported above.

This deep exploration of the quercetin and related flavonoid derivatives highlights the enthusiasm of therapeutic science specialists towards finding new potent and selective monoamine oxidase inhibitors or useful targeting agents for neurological and mental disorders. The current review is aimed to demonstrate the tremendous pharmacological MAO inhibition profile of natural flavonoid derivatives. The experimental in vitro studies suggested that natural flavonoids showed micro- to nanomolar range IC_50_ values against both MAO isoforms. Furthermore, the docking studies correlated in many experiments to explore the molecular mechanism of flavonoid at the MAO receptor level. This may give the idea for the structural activity requirement of different classes of natural flavonoids for the MAO inhibition. Compilation of overall SAR studied indicated some characteristics of flavonoid moiety (Figure 19). The glycosylation with sugar reduces the hMAO inhibitory potential of flavonoid as studies by Lee and coworkers [50]. Moreover, the mono-substitution enhance the selectivity towards hMAO-A; di-substitution enhance selectivity towards hMAO-B as indicated by Chimenti and coworkers [71]. The unsturation of chromone ring is crucial for MAO inhibition. Nevertheless, Presence of OH group decrease the MAO inhibitory potential as observed by Turkmenoglu and coworkers [72]. Hence, the perditions of in vitro and in silico properties on flavonoid moiety could help to further modification and clinical exploration as flavonoid based potent MAO inhibitors.

## Figures and Tables

**Table 1:** Docking profile of natural flavonoids with target protein and interactions.

Flavonoids	Target Protein	Important Amino Acid Residues	Comments	Software	References
Quercetin	MAO-A PDB (2Z5X)	Tyr444, Tyr197, and Asn181	Quercetin fitted well within the hMAO-A active site than in the hMAO-B active site due to development of highest *π*-*π* interaction and intermolecular hydrogen bonds.	Schrodinger [61]	Zhang et al. [62]
Baicalein	MAO-B PDB (2Z5Y)	Leu164 and Leu167	Two catecholic OH groups of baicalein showed hydrogen bonding with Leu167and Leu164 respectively.	Schrodinger [61]	Gao et al. [63]
6-prenyl apigenin	hMAO-A PDB (2Z5X)	Tyr 444 and Tyr407	6-prenyl apigenin the structural shared π electrons of the hydroxyl groups were sandwiched between phenolic side chains of TYR407 and TYR 444 composed the ‘aromatic cage’ of the hydrophobic pocket of the enzyme.	AutoDock [64]	Beula et al. [65]
Kaempferol	hMAO-A PDB (2Z5X)	Ile335 of hMAO-A Tyr326 of hMAO-B	Kaempferol in the dynamic site of hMAO-A established hydrophobic interactions with important residues of hMAO-A for a longer time than in the hMAO-B pocket.	Schrödinger [61]	Gidaro et al. [66]

**Table 2:** Docking profile of natural flavonoids from *Sideritis*, against MAO-A PDB (2Z5X) and MAO-B PDB (2XFU) with docking score and free binding energy (ΔG) and Ki (μM).

Sr. No	Flavonoid	Binding Score Energy Value for MAO-A (Kcal/mol)	Calculated Ki for MAO-A (μM)	Binding Score Energy Value for MAO-B (Kcal/mol)	Calculated Ki for MAO-B (μM)
1	Isoscutellarein 7-*O*-[6′′′-*O*-acetyl-β-d-allopyranosyl-(1→2)]-6″-*O*-acetyl-β-d-glucopyranoside	−3.81	1660.00	8.92	-
2	Salvigenin	−8.30	0.867	−7.51	3.63
3	Isoscutellarein 7-*O*-[6′′′-*O*-acetyl-β-d-allopyranosyl-(1→2)]-β-d-glucopyranoside	−4.15	930.10	5.79	-
4	Xanthomicrol	−7.80	1.90	−5.78	64.26

**Table 3:** Natural flavonoids docked against MAO-A PDB (2Z5X) showing docking score and free binding free energy (Kcal/mol) and inhibition constant Ki (µM) with no. of hydrogen bonds along with interacting amino acid residues.

Sr. No	Name of the Lead	Binding Free Energy (Kcal/mol)	Inhibition Constant Ki (µM)	No. of Hydrogen Bonds	Interacting Amino Acid Residue
1	Kaempferol	−5.17	4.63	12	397 TRP, 352 PHE, 406 CYS, 444 TYR, 448 ALA, 303 VAL, 51ARG, 407 TYR, 52 THR, 435 THR, 305 LYS, 445 MET
2	Quercetin	−4.40	636.60	9	436 GLU, 448 ALA, 52 THR, 435 THR, 407 TYR,51 ARG, 406 CYS, 23 ILE, 445 MET
3	Apigenin	−7.65	2.61	8	305 LYS, 397 TRP, 448 ALA, 51 ARG, 406 CYS, 435 THR, 352 PHE, 407 TYR
4	Luteolin	−7.67	2.42	11	448 ALA, 23 ILE, 435 THR, 406 CYS, 303 VAL, 52 THR, 51 ARG, 397 TRP, 445 MET, 407 TYR,444TYR
5	Brofaromine (Standard)	−7.55	3.06	10	303 VAL, 397 TRP, 51 ARG, 52 THR, 406 CYS, 305 LYS, 445 MET, 407 TYR, 435 THR, 448 ALA

**Table 4:** Inhibition affinity by docking studies, of MAO isoforms by flavanones of *Psoralea corylifolia* extract.

Sr. No	Natural Ligands	MAO-A Active Site PDB (2BXR)		MAO-B Active Site PDB (1GOS)		Overall Bonds		MAO Inhibition Selectivity
		Docking Score	Predicted H-Bond	Docking Score	Predicted H-Bond	H-Bond	Active Site Residue	
1.	Bavachinin	−1.06	0	−6.82	2	OH⋯O HO⋯HN	THR:201: A THR:201: A	B
3	Safinamide	−0.22	0	−6.12	3	NH⋯O NH⋯O NH⋯O	GLU:84: A THR:201: A PRO:102: A	B
4	Bavachin	−8.72	H_2_O-726	−3.95	0	⋯	⋯	NA

**Table 5:** Docking scores of isoflavone genistein and daidzein within human monoamine oxidase-A and -B binding sites.

Sr. No	Name of the Lead	MAO-A		MAO-B		RMSD Å	Amino Acid
		Docking Score	Predicted H-Bond	Docking Score	Predicted H-Bond		
1	Genistein (GST)	−7.0	0	−12.8	2 (OH⋅ ⋅ ⋅ N)	2.27	THR: 201: A
2	Daidzein (DZ)	−6.9	0	−12.8	1 (O⋅ ⋅ ⋅ HN)	2.32	THR: 201: A

**Table 6:** Summary of the molecular docking studies and inhibitory activity of compounds against MAO-A and MAO-B enzymes.

Sr. No	Name of the Lead	hMAO-B		hMAO-A	
		IC_50_ (μM)	ΔG Bind (Kcal/mol)	IC_50_ (μM)	ΔG Bind (Kcal/mol)
1	Kaempferol	>100	−42.66	0.525 ± 0.035	−49.52
2	Quercetin	>100	−46.98	3.98 ± 0.265	−48.35
3	Harmine	-	-	0.029 ± 0.0042	−46.07
4	Safinamide	0.0479 ± 0.00472	−73.70	-	-
